# Employing emerging technologies such as motion capture to study the complex interplay between genotype and power-related performance traits

**DOI:** 10.3389/fphys.2024.1407753

**Published:** 2024-05-22

**Authors:** Ioannis Papadimitriou

**Affiliations:** Department of Physiology, Faculty of Science, Mahidol University, Bangkok, Thailand

**Keywords:** cross-field, genomics, physiology, biomechanics, epigenetics

## Abstract

Exercise genomics has progressed alongside advancements in molecular genetic technologies that have enhanced our understanding of associations between genes and performance traits. This novel field of research incorporates techniques and tools from epidemiology, molecular genetics, exercise physiology and biostatistics to investigate the complex interplay between genotype and specific quantitative performance traits, such as muscle power output. Here I aimed to illustrate how interdisciplinary training can ensure the effective use of new emerging technologies, such as motion capture, to examine the influence of genetic and epigenetic factors on power-related quantitative performance traits. Furthermore, this study raises awareness about the present research trends in this field, and highlights current gaps and potential future developments. The acquired knowledge will likely have important future implications in the biotech industry, with a focus on gene therapy to combat age-related muscle power decline, personalized medicine and will drive advancements in exercise program design.

## Introduction

**FIGURE 1:**
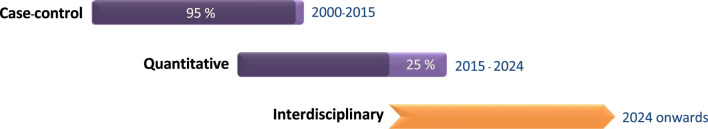
The timeline of different types of research conducted in the field of exercise genomics, as well as the future direction.

The ground-breaking polymerase chain reaction (PCR) method was invented by Kary Mulis in 1983, and in the decades since that discovery, remarkable advancements have been made in genetic research. Cutting-edge molecular technologies have refined our approach to investigating how genetic predisposition influence performance traits. Innovative genetic research tools led to the formal recognition of the field of exercise genomics in the late 2000s. Initial research primarily focused on common genetic polymorphisms,^1^ with studies mainly using the case-control approach, without incorporating quantitative performance measures as relevant biomarkers (Woods et al., 2001; Yang et al., 2003; Papadimitriou et al., 2008; Papadimitriou et al., 2009; Mikami et al., 2013). Subsequent molecular genetic research enabled us to investigate the quantitative contribution (Papadimitriou et al., 2016; Contrò et al., 2018; Papadimitriou et al., 2018; Wei, 2021), gain genetic insights into physical characteristics, such as muscle strength (Clarkson, 2005; Broos et al., 2015; Willems et al., 2017) and explore the mechanistic pathways (Papadimitriou et al., 2019) that explain the interactions between genes and exercise training Figure 1.

While these methods have shown some effectiveness (Papadimitriou et al., 2016; Willems et al., 2017; Contrò et al., 2018), their implementation to explore complex performance characteristics—such as speed and muscle power—has proven to be more challenging (Hanson et al., 2010; Ruiz et al., 2011; Broos et al., 2015). One possible explanation is that the contributions of common genetic polymorphisms to these performance characteristics typically have small effect sizes, and these approaches lack the sensitivity required to detect such minor effects. Furthermore, considering that the majority of athletic outcomes involve multi-joint movements, the suitability of a number of the technologies that were previously employed may be questioned. Moreover, complex quantitative traits commonly exhibit further epigenetic influence from factors that regulate gene expression, such as DNA methylation (Raleigh, 2012).

Determining how common genetic polymorphisms influence athletic performance and human locomotion is challenging for several reasons—including the difficulty of accurately defining precise biomarkers; challenges in identifying or measuring the influences of environmental, epigenetic, and anthropometric factors; inadequate sample sizes; and technological limitations in obtaining sufficient and high-quality genetic and performance data. Rapid advancements in technology have yielded new and improved research tools that enable us to overcome these limitations. These tools enhance the accuracy and specificity of measurements, and improve the quality of created databases. These advancements enable us to more efficiently and precisely identify and quantify how common genetic variants and epigenetic factors influence physical performance.

Exercise physiologists now have access to several new emerging technologies, as it will be discussed in the paper. These include motion capture, which enhances the accuracy and specificity of performance measurements, bead-based DNA isolation methods that yield efficiently high-quality DNA, and novel microarray-based assays that enable rapid measurement of large numbers of genetic and epigenetic markers.

Motion capture technology enables the precise detection and analysis of specific performance characteristics, such as torque and velocity. Initially, this technique was used in life sciences for gait analysis. Today, motion capture is utilized in additional fields, including neuroscience and robotics. As motion capture systems continue to improve and expand, they hold great potential for applications in exercise genomics (Htet et al., 2023) and future use in epigenetics.

To fully enable the potential of today’s highly skilled exercise physiologists and sport geneticists, we must support them in integrating the latest technologies, with emphasis on the crucial role of interdisciplinary research.

## Motion capture technology in sport genomics: a guide for incorporating an emerging technology

Over the past 50 years, a wide range of vision-based methods have been developed to track human movement. An in-depth review of these approaches is available in the work of (Moeslund et al., 2006). Here, I aim to provide a brief overview of the evolution of the techniques used in the field, with an emphasis on the potential for revolutionized future applications in genomic and epigenomic research. The differences between these systems lie in the camera configuration, representation of recorded data, and types of tracking algorithms employed (Lee and Chen, 1985; Narayanan et al., 1995; Kakadiaris and Metaxas, 1998; Wagg and Nixon, 2004).

While there are notable differences in the technical characteristics of these techniques (Richards, 1999), they all share the same fundamental concept. They involve identifying spots of interest in sequential image frames, converting them into real-space coordinates, and using this information to determine the three-dimensional (3D) position of the visualizing skeleton (Corazza et al., 2006; Baran and Popović, 2007).

Prior to the introduction of digital technology, film analog cameras were commonly employed for tracking human movement during athletic activities (Procter and Paul, 1982; Bobbert et al., 1986; Lees et al., 1993). Some of this technology proved helpful in analyzing explosive body movements due to its greater resolution and enhanced recording frequency. However, the effectiveness of this approach was hindered mainly by lengthy processing times and complex data interpretation.

Over the last decade, there has been a remarkable progress in the study of human movement. This is largely due to the utilization of cutting-edge optoelectronics (Windolf et al., 2008). Faster computing power, larger memory, and hardware shrinkage have further facilitated these advances. Due to these exciting developments, research methodologies made significant improvements and a plethora of innovative techniques of automatic recognition of body movements have been developed. Most of these systems employ numerous cameras that emit infrared radiation, together with reflective markers that reflect this radiation back to the cameras. This enables the recording devices to determine the 3D position of the markers fast and accurately (Mündermann et al., 2006; Maletsky et al., 2007). Recent technological advancements have even made it feasible to detect motion without the need for reflective markers (Saini et al., 2015). These marker-less techniques often have much faster processing times and an improved recording range. However, they demonstrate reduced accuracy and precision, making them unsuitable for research applications (Klous et al., 2010). Currently, marker-based optoelectronic measurement systems are often regarded as the gold standard in motion capture. They outperform all other technologies in terms of the accuracy and precision required for prospective usage in research environments (Corazza et al., 2010; McGuirk et al., 2022; Johnson et al., 2023).

While motion capture is already prevalent in the life sciences industry, there remains potential for growth in research settings including in the fields of exercise genomics, physiology, and epidemiology. There is great demand for highly accurate motion capture technology in research. Current advancements in hardware have overcome previous limitations, but for this potential to be realized, it is important to educate and raise awareness among researchers who may have little experience working with motion tracking data. In particular, exercise physiologists and geneticists can benefit from using this high-quality data in their investigations. Ultimately, to fully harness the advantages of motion capture technology in research, we must increase acceptance and understanding among a range of researchers and their respective fields.

Moreover, motion capture alone may not be sufficient to qualify the performance levels of individuals. To successfully qualify and quantify the genetic and epigenetic influences it is necessary to evaluate dynamic performance characteristics, such as ground reaction forces and the rate at which force is developed incorporating additional technologies such as force plate dynamometry.

My research team integrated motion capture technology with force plate dynamometry in hopes of identifying novel power-related outcomes that are more sensitive to allele-specific differences. The study we conducted presents evidence that there is a connection between allele-specific differences and specific performance characteristics in certain joints during explosive body motions, such as sprinting or jumping (Htet et al., 2023). The physiological and biomechanical parameters identified from these analyses are shown in Table 1. These variables can serve as biomarkers in future studies to reveal subtle associations between performance traits and genetic or epigenetic factors.

**TABLE 1:** The table provides a summary of the most relevant physiological and biomechanical parameters that arise from these techniques and that can be linked to genetic and epigenetic factors along with their calculation formulas.

	Variables	Formulas	Technology	References
Angular	Relative torque	$RT=\frac{Segmental\;mass\left(kg\right)*Angular\;acceleration\;\left(\deg s^{-2}\right)}{Total\;body\;mass\left(kg\right)}$	Motion capture	Knudson (2007)
	Power	$P=Relative\;torque\left(N_{m}\right)*Angular\;velocity\left(\deg s^{-1}\right)$		Sayers et al. (1999)
Dynamic	Peak power	$PP=Force\;at\;peak\;velocity\;\left(N\right)*Peak\;velocity\left(ms^{-1}\right)$	Integration of motion capture systems with force plate dynamometry	Turner et al. (2012)
	Rate of force development	$RFD=\frac{Peak\;Force\left(N\right)}{Time\;to\;peak\;Force\left(s\right)}$		Comfort et al. (2011)
Reactive	Reactive strength index	$RSI=\frac{DJ\;Height\left(m\right)}{Ground\;Contact\;Time\left(s\right)}$		Riggs and Sheppard (2009)
	Index of reactive force	$IReaF=\frac{DJ\;Height-SJ\;Height\;\left(m\right)}{SJ\;Height\left(m\right)}$		Papadopoulos et al. (2009)

The angular parameters quantify relative torque and power during explosive body movements, such as jumps and sprints, as recorded by motion capture technology. On the other hand, the dynamic variables represent power related characteristics of the force-time curve as measured by force plate dynamometry. Furthermore, the reactive parameters quantify the elastic utilization during vertical jumps with very short ground contact time, and increased muscle contraction velocity combining an explosive coupling between an eccentric and concentric muscle action, commonly known as stretch-shortening cycle (SSC) (Nicol et al., 2006). These parameters are determined based on participants’ performance in Squat Jump (SJ) and Drop Jump (DJ), which was measured using motion capture technology (Htet et al., 2023).

Motion capture technology allows sport geneticists and exercise physiologists to incorporate biomechanical biomarkers into studies of allele-specific performance traits, thereby improving the accuracy and specificity of the measurements. The potential to include a biomechanical marker that reflects the genetic effect on a specific joint represents an advancement compared to traditional approaches for assessing speed and power-related performance in genetic research.

Compared to previous research approaches, the potential widespread utilization of motion capture technology in sport genomics offers several advantages. Firstly, it enables a focus on specific joints without influence by the overall anthropometric parameters of the human body. Secondly, it facilitates the analysis of full-body movements. Furthermore, it enables detailed analysis of more specific performance characteristics, such as peak torque and velocity, with regards to individual joints.

Before motion capture technology can be implemented in exercise genomic research, exercise physiologists must thoroughly assess the characteristics and reliability of this new laboratory approach. This requires analyzing the sensitivity, specificity, and other aspects that could affect the accuracy of the measurement. A series of preliminary and validation studies must be conducted (Moe et al., 2022) before these biomarkers can be used in large-scale genetic investigations. For example, researchers must determine how much of the variation in allele-specific measurements can be attributed to individual differences, intra-individual variability, or laboratory error. Each stage of this investigation process requires a foundation in exercise genomics, as well as expertise in various academic fields, potentially including physiology, biomechanics, and biostatistics. Thus, a sport geneticist or an exercise physiologist must have a comprehensive understanding and involvement in all components of the study, even those that extend beyond traditional exercise physiology or genomic training.

## Other emerging technologies with potential application in the field

### Bead-based DNA separation methods

Microarrays have been extensively utilized as platforms for SNP detection (Bumgarner, 2013). These technologies require high quality sample preparations, and the process of DNA isolation for sport genomic research continues to be a time-consuming task, relying on various extraction and centrifugation steps (Head et al., 2014).

Magnetic DNA isolation is an emerging technology that utilizes the power of magnetism for fast and effective DNA extraction and purification (Haddad et al., 2016). Column-based procedures involve the centrifugation of the lysate, followed by the addition of the supernatant to a silica membrane for the purpose of binding nucleic acids. The DNA is then washed and finally released in an adequate volume of elution buffer. These steps present a significant risk of causing mechanical damage to the DNA. Magnetic bead-based procedures have a reduced number of handling stages, and certain protocols eliminate the need for centrifugation, which reduces the risk of shearing compared to column-based methods (Chacon-Cortes and Griffiths, 2014; Ali et al., 2017). This improvement in efficiency also leads to lower costs.

Exercise geneticists can benefit from using this high-quality DNA in their studies, but in order to take full advantage of this potential, it is crucial to promote awareness among researchers who might lack familiarity working with this methodology.

### DNA micro-array based methylation assays

In recent years, there has been growing interest in health-related fitness epigenetics. Complex power-related quantitative traits commonly exhibit varying degrees of genetic influence, as well as further influence from the epigenome. The epigenome serves as the interface connecting the environment with the genome.

Epigenetic modifications involve changes of gene expression that occur without any alternations to the DNA sequence and include DNA methylation, histone modifications, and microRNA expression (Ling and Groop, 2009). An explanation of all types of epigenetic modifications is beyond the scope of this mini review study and may be obtained elsewhere (Portela and Esteller, 2010). Here I aim to raise awareness and understanding of newly developed technologies employed in research examining DNA methylation and their potential application in exercise genomics.

DNA methylation involves the attachment of a methyl group to the 5-carbon position of the pyrimidine nucleotide cytosine. This mark predominantly appears within the context of a CpG dinucleotide (Brait et al., 2008). This kind of regulation changes the gene expression by functioning as a bidirectional valve. Once a particular CpG reaches a specific CpG location, it leads to the suppression of gene expression, whereas its demethylation facilitates gene expression.

In order to investigate this process, several laboratory techniques have been created to assess different aspects of DNA methylation (Laird, 2003). The utilization of these methods originated in the field of cancer research and includes global methylation, which characterizes an individual’s general methylation profile (Zhang et al., 2011), and methylation that is specific to a particular gene, which regulates how certain genes are expressed (Deneberg et al., 2010).

The introduction and extensive use of epigenome-wide DNA methylation technologies—such as Illumina Infinium Human Methylation 450K assay—allowing rapid and simultaneous measurement of methylation levels at around 480,000 CpG sites across the genome (Wang et al., 2015), led to the development of DNA methylation-based biomarkers for various types of health related fitness characteristics (Yousefi et al., 2022; Jokai et al., 2023). Biomarkers of methylation levels may have future applications in large-scale studies to assess the complex relationships between genotype, gene expression, and specific quantitative performance characteristics such as muscle torque and power.

## Discussion

This work highlights the potential for a future utilization of novel biomarkers and technologies in the context of exercise genomic research. With the growing acceptance of emerging technologies, such as motion capture, there is a significant opportunity to improve our understanding of the relationship between common genetic variants and certain quantitative performance traits, such as muscle power output, as well as identify the potential influence of epigenetic factors.

Most quantitative genetic studies have demonstrated that specific common genetic variants typically account for only about 2%–3% of the variation in muscle speed and power performance (Papadimitriou et al., 2016). The remaining variability is influenced by a diverse array of genetic, epigenetic, and environmental factors—the majority of which are not well understood, especially the genetic and epigenetic factors.

The presently available data support the conclusion that the genetic predisposition to power-related performance traits and responses to physical training are determined by the interplay of numerous genes and non-genetic factors, rather than by a single gene or few alleles. Elucidating the effects of common genetic variations on human movement and athletic performance is a difficult task. Challenges include the precise detection of common variations in DNA, the determination and quantification of anthropometric and epigenetic variables, technological constraints in procuring genetic and performance data of sufficient quantity and quality, and the complex characteristics inherent in conducting research with humans.

Future research in the field of exercise genomics should be composed of a larger amount of functional quantitative research and a smaller fraction of case-control studies as illustrated in Figure 1. Case-control studies have played a key role in the initial stage of exercise genomics research, as they constitute a useful approach for investigating the association between common genetic variations and athletic performance. However, they have several drawbacks, including being prone to bias, and the majority of published candidate gene studies suffer from limited sample numbers and insufficient statistical power. Therefore, it is important to confirm the findings of case-control studies through replication with improved quantitative research designs (Papadimitriou et al., 2016; Contrò et al., 2018).

The potential use of motion capture technology in sport genomics provides several advantages compared to earlier research analyses in this field (Moran et al., 2007; Ruiz et al., 2011). Firstly, it enables a focus on specific joints without being affected by general anthropometric factors of the human body. Furthermore, it facilitates a comprehensive full-body examination of specific performance characteristics, including the peak torque and power generated at individual joints during explosive body movements. Such methodological and technological advancements have facilitated the identification of more precise phenotypes, and improved study designs for detecting and measuring the effects of common genetic variants on complex phenotypes, such as those associated with muscle speed and power (Htet et al., 2023).

Further research is necessary to expand beyond common genetic variants, combining transcriptomics with genomics and utilizing motion capture technology to investigate the potential influence of DNA methylation markers on training adaptations in specific performance characteristics important for speed and power.

Moreover, motion capture alone may not be sufficient to qualify the performance levels of individuals. Integrating additional technologies like force plate dynamometry is crucial. Muscular, body related signals and cerebral activities are also essential elements to be able to refine the intrinsic quality of the movement and today accompany the measurement of movement in the field (Cosendey et al., 2023).

Future exercise geneticists must thoroughly understand diverse disciplines to determine the appropriate use of emerging technologies and novel biomarkers, to assess their validation, and to ensure their meaningful interpretation. In a way, an exercise geneticist can be viewed as the stage director of a scientific project, which includes physiologists, biomechanical research scientists, computer vision analysts, laboratory technicians and bioinformatics experts. Just as it is impractical for a stage director to be involved in every role in a theater performance, it is also unrealistic for an exercise geneticist to be an expert in every role required for modern genomic research. However, like a stage director, an exercise geneticist must have a thorough understanding of each discipline, in order to coordinate and synthesize their contributions. Thus, in addition to expertise in genomic research, an exercise geneticist must also have a multidisciplinary skill set. Embracing interdisciplinary education in exercise genomics will cultivate an appreciation for different perspectives and methodologies within traditional fields of study and will facilitate the effective utilization of new emerging technologies to shed light on complex interplay between genome, epigenome and power-related performance traits.

This integrated knowledge has the potential to greatly impact the biotech industry, particularly in terms of age-related muscle power decline, and future applications in personalized exercise training programs, that aim to tailor training based on an individual’s genetic makeup (Amato et al., 2018). Overall, this field of research can greatly enhance human health and well-being.

Lastly, incorporating rapidly emerging technologies can be a daunting task due to the overwhelming amount of data they produce. Prior to conducting further research in the field, it is imperative to employ discovery-based strategies to sort through the vast amount of data generated and pinpoint specific markers. This challenge led to emergence of the bioinformatics field, and it is now essential to have a biostatistician or an expert in data interpretation as a collaborator. Furthermore, artificial intelligence has the capacity to discover novel approaches to analyze biomarker and genomic data, equipping us with cutting-edge tools for predicting genes or epigenetic factors associated with performance traits and to address challenges associated with evaluating and understanding vast quantities of genomic and biomechanical data (Nguyen et al., 2022). Moreover, the large quantity of data being generated fundamentally changes the validation process, which now necessitates both discovery and hypothesis testing.

In a nutshell, exercise geneticists can greatly benefit from incorporating emerging technologies, like motion capture, into their studies. However, to fully realize this potential, it is crucial to raise awareness while improving acceptance and comprehension among researchers from various fields.

## Footnotes

1 Common genetic polymorphisms, most often single-nucleotide polymorphisms (SNPs), are heritable nucleotide differences between individuals that occur in at least 1% of the population (Karki et al., 2015).
